# Oleic Acid Facilitates Cd Excretion by Increasing the Abundance of *Burkholderia* in Cd-Exposed Mice

**DOI:** 10.3390/ijms232314718

**Published:** 2022-11-25

**Authors:** Zhijia Fang, Yinyan Chen, Yongbin Li, Lijun Sun, Qi Deng, Jingwen Wang, Ravi Gooneratne

**Affiliations:** 1 College of Food Science and Technology, Guangdong Ocean University, Zhanjiang 524088, China; 2 Guangdong Provincial Key Laboratory of Aquatic Product Processing and Safety, Zhanjiang 524088, China; 3 Guangdong Provincial Engineering Technology Research Center of Marine Food, Zhanjiang 524088, China; 4 Key Laboratory of Advanced Processing of Aquatic Products of Guangdong Higher Education Institution, Zhanjiang 524088, China; 5 Department of Wine, Food and Molecular Biosciences, Lincoln University, Lincoln, Canterbury 7647, New Zealand

**Keywords:** cadmium accumulation, cadmium excretion, oleic acid, gut microbiota, *Burkholderia cepacia*, extracellular polymeric substances

## Abstract

As a global pollutant, cadmium (Cd) can easily enter the body through food chains, threatening human health. Most Cd is initially absorbed in the gut, with the gut microbiota playing a pivotal role in reducing Cd absorption and accumulation. This study assessed the effects of three fatty acids on Cd accumulation and toxicity in Cd-exposed mice. The results showed that oleic acid (OA) was the most effective in facilitating Cd excretion in mice among these fatty acids. The use of OA led to reduced Cd accumulation in the organs and increased Cd content in the feces. The metagenomic analysis of the gut microbiota showed that the genus *Burkholderia* was the most significantly restored by OA in Cd-exposed mice. *Burkholderia cepacia*, as the type species for the genus *Burkholderia*, also exhibited strong Cd tolerance after treatment with OA. Furthermore, the electron microscopy analysis showed that most of the Cd was adsorbed on the surface of *B. cepacia*, where the extracellular polymeric substances (EPSs) secreted by *B. cepacia* play a key role, displaying a strong capacity for Cd adsorption. The peak at 2355 cm^−1^ and the total sulfhydryl group content of EPSs showed significant increases following co-treatment with Cd and OA. The results demonstrated the potential roles that gut *Burkholderia* may play in OA-mediated Cd excretion in mice.

## 1. Introduction

As a global food contaminant, cadmium (Cd) can easily enter and accumulate in the human body [1,2]. Prolonged exposure to Cd will produce adverse health effects due to its accumulation [3]. Cd accumulation in the body can lead to multiple organ damages and health diseases, including cancers [4,5]. Most accumulated Cd is initially absorbed in the gut before being transported to other organs [6,7]. However, it would be difficult to eliminate Cd in vivo at short notice. The accumulative property of Cd may be ascribed to its high affinity to endogenous substrates, such as protein and DNA [8], and its low excretion rate [9]. Thus, the promotion of Cd excretion and reductions in Cd accumulation in the body have been hot areas of research for several years.

Several physical and chemical methods have been used for the removal of Cd from wastewater [10]. However, it would be unfeasible to promote Cd excretion from the human body via these physical and chemical methods due to their inherent drawbacks in terms of safety. Fortunately, evidence has shown that lipidic supplements exert a particular reductive effect against Cd accumulation [11,12]. Our previous study reported that oleic acid (OA) could significantly reduce Cd-induced multiple tissue damage, particularly in the liver and kidneys [13]. Additionally, changes in the lipidic content and fatty acids profiles were examined upon Cd exposure, and supplemental fatty acids were beneficial for mitigating Cd poisoning [14,15]. However, the reductive mechanism of fatty acids on Cd accumulation in the body is still unclear.

Cd is primarily absorbed through the gut [16,17]. The gut microbiota affects the heavy metal absorption and metabolism [18]. The gut microbiota plays a pivotal role in Cd absorption and accumulation [19]. Cd exposure can change the intestinal microbial composition [20] and destroy the gut microbial diversity [21]. In Cd-exposed mice, an impaired gut barrier can exacerbate Cd accumulation in multiple organs (liver, kidneys, and small intestine) [17]. Even worse, Cd can perturb the gut microbiota communities, aggravating the Cd accumulation and hindering Cd excretion [22]. The gut microbiota is considered a toxicological indicator in response to stress from environmental pollutants, such as Cd [23]. Moreover, the role of the gut microbiota as a protective factor against Cd accumulation in the liver, spleen, and kidneys of mice was indicated in an earlier study [24]. The oral administration of gut microbiota, such as *Akkermansia muciniphila*, could lead to reduced Cd toxicity [25]. On the other hand, Cd exposure altered the bacterial metabolism and accelerated oleic oil production in *Caenorhabditis elegans* [26]. According to a new study, dietary fatty acids can sustain the composition of gut microbiota [27]. Alpha-linolenic acid (ALA) has a significant regulatory effect on the gut microbiota of mice fed a high-fat diet [28]. In addition, dietary linoleic acid (LA) altered fecal microbiome diversity before and during pregnancy [29]. It has been hypothesized that these fatty acids could exert their reductive effects on Cd accumulation by sustaining some key members of the gut microbiota.

The aim of this study was to clarify the reductive mechanism of fatty acids on Cd accumulation in the body from the perspective of the gut microbiota. In this study, the effects of fatty acids on Cd accumulation and excretion were assessed. Furthermore, their effects on the gut microbiota were evaluated, and the key gut microorganism contributing the most to the reduction in Cd accumulation was identified. Eventually, the Cd-adsorption behavior of the key gut microorganism was characterized. Overall, this study may potentially provide a reliable and safe means for reducing Cd accumulation in the body.

## 2. Results

### 2.1. Effects of Fatty Acids on Cd Excretion in Cd-Exposed Mice

**Figure 1:**
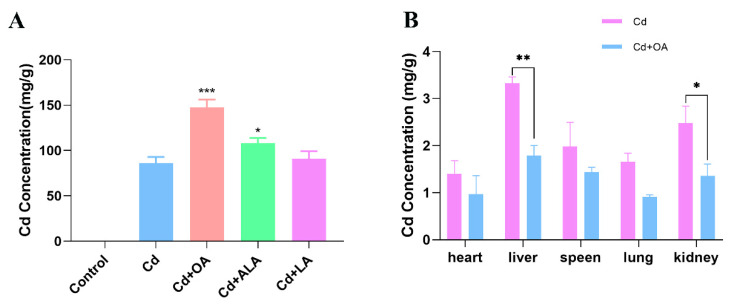
Effects of different fatty acids on the Cd contents in Cd-exposed mice. (**A**) The Cd contents in feces of Cd-exposed mice treated with three kinds of fatty acids for 7 days. (**B**) The Cd contents in different organs of Cd-exposed mice treated with OA. * *p* < 0.05, ** *p* < 0.01, and *** *p* < 0.001 versus the Cd group.

To compare the promotive effects of 3 fatty acids (OA, linoleic acid (LA), and alpha-linolenic acid (ALA)) on Cd excretion, the Cd levels in feces and organs in Cd-exposed mice after the oral administration of fatty acids were determined. The three fatty acids significantly increased the fecal Cd levels to varying degrees, and OA was the most effective in promoting Cd excretion (*p* < 0.001). Compared with the Cd group, the Cd concentrations in the feces of the mice in the OA-treated group increased by 71.61%, indicating that OA markedly enhanced the excretion of Cd via feces (Figure 1A). On the other hand, the Cd levels in multiple organs were significantly decreased after 7 days of oral administration with OA, particularly in the liver and kidneys (*p* < 0.05) (Figure 1B), suggesting that OA could abate Cd accumulation in the organs by promoting Cd excretion in the feces.

### 2.2. Effects of Oleic Acid (OA) on Gut Microbiota and B. cepacia against Cd

**Figure 2:**
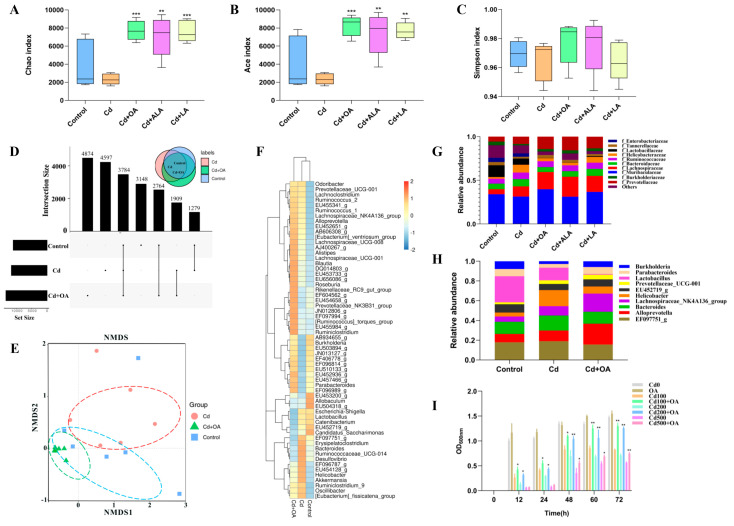
Effects of OA on the gut microbiota. The Alpha diversity of the gut microbial community: Chao (**A**), Ace (**B**), and Simpson (**C**) index. (**D**) The OTUs results at 97% identity level in UpSet diagram of the control, Cd−treated, and OA−treated groups. (**E**) The Beta diversity of the gut microbial community by NMDS maps. (**F**) The microbial abundance heatmap (log10) at the genus level. (**G**) The relative abundance of the top 10 gut microbiota at the family level. (**H**) The relative abundance of the top 10 gut microbiota at the genus level. (**I**) The growth of *B. cepacia* cultivated in LB broth containing 0, 100, 200, and 500 mg/L of CdCl_2_ and 2 mmol/L of OA. * *p* < 0.05, ** *p* < 0.01, and *** *p* < 0.001 versus the corresponding Cd groups.

The effects of the fatty acids on the gut microbiota were analyzed using high-throughput 16S rRNA sequencing. Both microorganism community richness indices Chao and Ace of the gut microbiome significantly decreased after exposure to Cd (Figure 2A−C). In contrast, these two indices were restored following fatty acid supplementation (*p* < 0.05). However, the Simpson index indicated no significant differences between these five groups.

The numbers of shared OTUs of gut microbes at the 97% identity level in the UpSet diagram were compared between the groups. From the results shown in Figure 2D, the number of shared OTUs between the OA−treated and control groups was higher than that between the Cd−treated and control groups. In addition, the non−metric multidimensional scaling analysis (NMDS) in Beta diversity also showed that the overlapping regions between the OA−treated and the control groups were more extensive than that between the Cd−treated and control groups (Figure 2E). The results of both the UpSet diagrams and NMDS maps revealed that the structure of the gut microbiota in the OA−treated group was closer to the control group, accounting for the effect of OA on Cd toxicity, which the OA effectively restored.

To identify the critical gut microbes that contributed to the OA−mediated Cd excretion, the relative abundances of gut microbes at the family level in each group were compared. As shown in Figure 2F,G, compared to the control group, there were lower abundances of Muribaculaceae, Lactobacillaceae, Tannerellaceae, and Burkholderiaceae in the Cd group. However, the gut microbiota variations had been ameliorated due to the OA treatment, such as Muribaculaceae, Bacteroidaceae, Helicobacteraceae, Burkholderiaceae, and Tannerellaceae, among which Burkholderiaceae increased the most (79.77%). These results demonstrated that the OA significantly affected the abundance of gut microbiota inhibited by Cd, particularly Burkholderiaceae. Furthermore, the gut microbiota composition of the top 10 members at the genus level showed that *Burkholderia* from the Burkholderiaceae family increased most dramatically following OA supplementation (Figure 2H). *B. cepacia* has been described as the type species of the *Burkholderia* genus [30], and *B. cepacia* was reported to be as efficient for the bioaccumulation of Cd [31,32].

To confirm the effect of OA on *B. cepacia* in vitro, a standard strain *B. cepacia* (ATCC 25416) was employed in a Cd−sensitivity test (Figure 2I). Compared to the Cd group, the inhibited growth of *B. cepacia* cells was significantly restored by OA. Therefore, it can be concluded that OA could confer strong Cd resistance to *B. cepacia*, which may enable the restoration of the abundance of *Burkholderia* in Cd−exposed mice.

### 2.3. B. cepacia Facilitates Cd Excretion and Reduces Cd Accumulation in Cd-Exposed Mice

**Figure 3:**
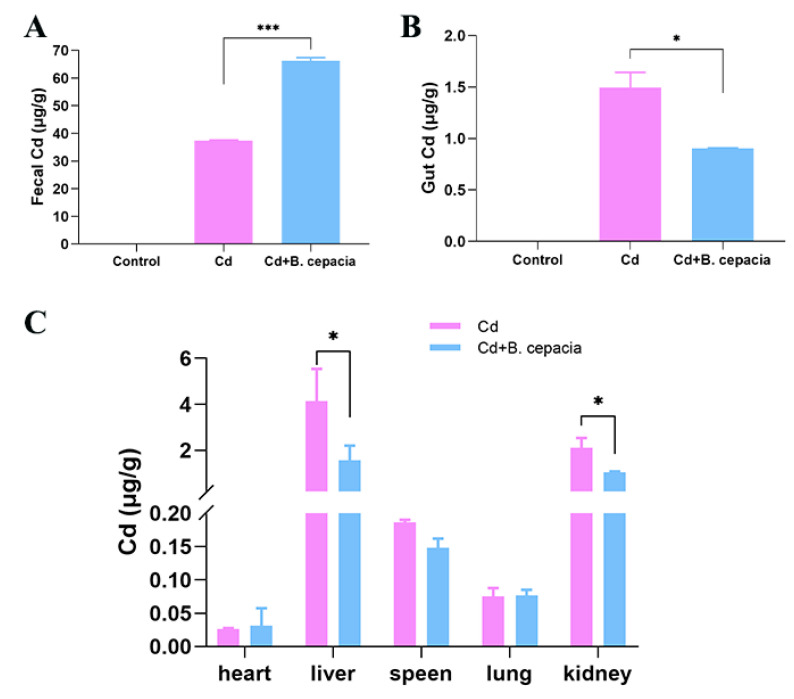
Cd accumulation in Cd-exposed mice. (**A**) Total Cd content in feces; (**B**) Cd content in the gut; (**C**) Cd contents in the heart, liver, spleen, lung, and kidneys after treatment for 7 d. * *p* < 0.05 and *** *p* < 0.001 versus the Cd group.

To assess the contribution of *B. cepacia* in the Cd excretion, *B. cepacia* was orally administrated to Cd-exposed mice, and the Cd contents in the feces, gut, and other organs were determined using ICP-MS. As shown in Figure 3, compared with the Cd group, the oral administration of *B. cepacia* resulted in the Cd levels significantly increasing in the feces but decreasing in the gut, liver, and kidneys. However, there was no significant change in the heart or lung tissues. These results demonstrated that *B. cepacia* plays an active role in reducing the residual tissue Cd contents while facilitating Cd excretion.

### 2.4. The Cd Content in B. cepacia and on Its Surface

**Figure 4:**
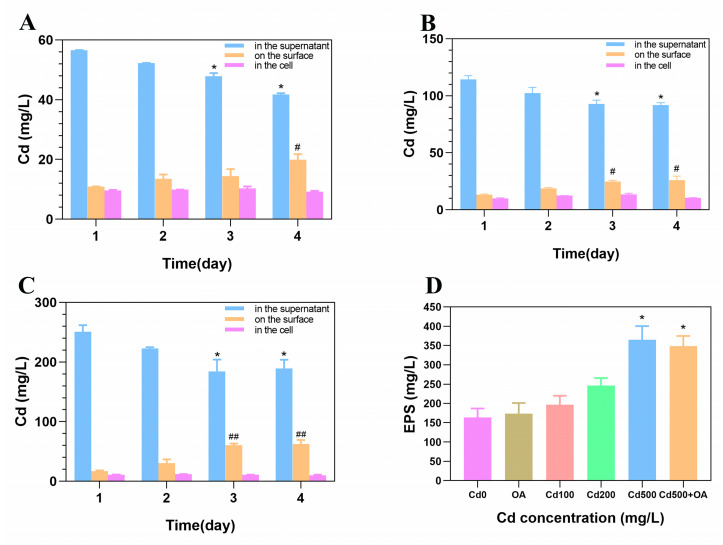
Cd bioaccumulation and production of EPS in *B. cepacia* after being exposed to (**A**) 100 mg/L, (**B**) 200 mg/L, and (**C**) 500 mg/L of CdCl_2_. * *p* < 0.05, ^#^ *p* < 0.05, and ^##^ *p* < 0.01 versus corresponding group on day 1. (**D**) EPS production of B. cepacia after being exposed to 0, 100, 200, and 500 mg/L of CdCl_2_ and 2 mmol/L of OA. * *p* < 0.05 versus the Cd 0 group.

To clarify the role of *B. cepacia* in facilitating Cd excretion, the amount of Cd in the cell and on its surface was assessed. As shown in Figure 4, the Cd content in the supernatant gradually decreased as the cultivating time was extended. In contrast, the Cd content on the surface of *B. cepacia* showed an expected increase. Moreover, after being exposed to 500 mg/L of Cd for four days, less than 4% of Cd was absorbed intracellularly, and about 24% was adsorbed on the surface. When the concentration of Cd increased, the amount of total extracellular polymeric substances (EPSs) gradually increased. Compared to the Cd-free group, the EPSs in Cd 500 showed a significant increase (*p* < 0.05), which may be attributed to the increase in Cd content on the surface of *B. cepacia*. However, the OA did not further increase the EPS production under Cd exposure. These results suggested that extracellular adsorption and intracellular uptake occur for *B. cepacia* exposed to Cd, but extracellular adsorption plays a dominant role.

### 2.5. Analysis of the Cd Distribution in B. cepacia Using Electron Microscopy

**Figure 5:**
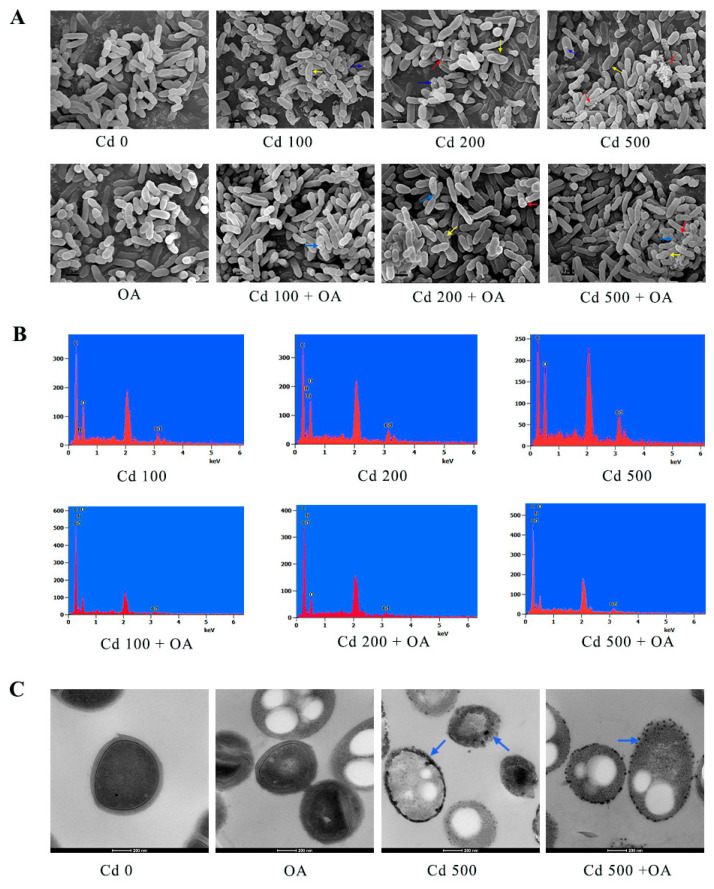
SEM (**A**), EDS (**B**), and TEM (**C**) images of B. cepacia under Cd exposure as well as co-treatment with Cd+OA. The EPS, Cd, and cell depressions are indicated by the red, blue, and yellow arrows, respectively. The Cd precipitates adsorbed on the cell surface and accumulated intracellularly are indicated by the blue arrows.

To monitor the adsorption of Cd by EPSs in *B. cepacia*, scanning electron microscopy (SEM) and transmission electron microscopy (TEM) were employed to characterize the cell morphology and Cd distribution. The plump and rod-shaped surface morphology for the *B. cepacia* cells was observed in the Cd 0 and OA groups, respectively (Figure 5A). After Cd exposure, significant depressions (as indicated by the yellow arrows) in the surface entrapped many cells, and these depressions became more severe with increasing Cd concentrations. In addition, EPSs and insoluble Cd particles (as indicated by the red and blue arrows, respectively) appeared on the cell surface, particularly in the Cd 500 group. In contrast, these surface depressions were relieved after the co-treatment of Cd and OA, exhibiting a relatively smooth surface compared to the Cd group. Meanwhile, the insoluble Cd particles and EPS-like flocculent materials were also observed in the Cd+OA groups. Cd was detected on the surface of *B. cepacia* exposed to Cd using an energy-dispersive spectroscopy (EDS) analysis (Figure 5B), which was confirmed by the TEM observation (Figure 5C).

### 2.6. Fourier Transform Infrared Spectroscopy (FTIR) Analysis of EPS Secreted by B. cepacia under Cd Stress

**Figure 6:**
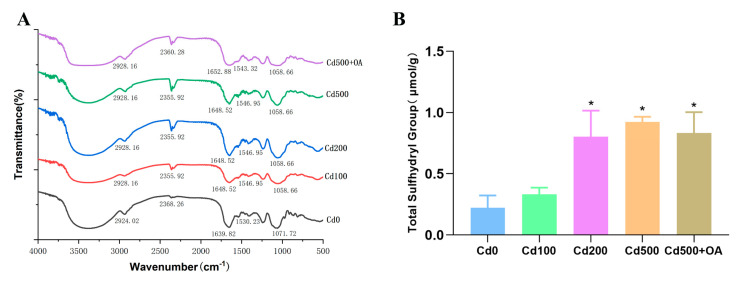
(**A**) FTIR spectroscopic analysis of EPSs produced by *B. cepacia* after being exposed to 0, 100, 200, and 500 mg/L of Cd and 2 mmol/L of OA. (**B**) The content of total sulfhydryl group in EPSs. * *p* < 0.05 versus control group.

FTIR spectroscopy was carried out to further reveal the Cd−adsorption mechanism of EPSs and to characterize functional group changes on the EPSs before and after Cd exposure. As shown in Figure 6A, the peak at 1058 cm^−1^ (P=O) increased with Cd and OA in co-treatment. The peaks at 1648 cm^−1^ (C=O and C=N) and 1530 cm^−1^ (N−H) were shifted after exposure to Cd [33,34,35]. Notably, the characteristic peak at 2355 cm^−1^ (−SH) increased in the presence of Cd and OA, suggesting an interaction between Cd and the P=O or −SH groups of EPSs.

The sulfhydryl group of EPSs usually acts as the binding site for metals [36]. The total sulfhydryl group content in the EPSs confirmed the −SH group changes due to Cd. As shown in Figure 6B, the total sulfhydryl group of the EPSs in *B. cepacia* significantly increased after 200 or 500 mg/L of Cd exposure. However, the OA did not further increase the total sulfhydryl group in the EPSs. The result suggests that Cd rather than OA increases the sulfhydryl content in EPSs, which may contribute to the enhanced Cd adsorption capacity of *B. cepacia*.

## 3. Discussion

Cd accumulation in the body is an urgent problem to be solved. OA was reported to possess good anti-Cd activity [13]. The gut microbiota plays the key role in Cd absorption and accumulation. In this study, we found that fatty acids, particularly OA, increased the gut levels of *Burkholderia*, and facilitated Cd excretion.

### 3.1. OA Reduced the Cd Content by Facilitating Cd Excretion

As an essential food supplement, fatty acids usually act as an important means of defense against Cd stress [37,38]. A high-fat diet was found to decrease hepatic iron storage by affecting iron absorption [39]. *Anethum graveolens* seed oil was found to reduce Cd deposition in the liver of Cd-exposed mice [40]. In the present study, OA showed a marked Cd detoxification, increasing fecal Cd content while significantly reducing the Cd levels in the liver and kidneys. This result is in line with an earlier report, in which a high-fat diet effectively reduced heavy metal accumulation in the liver and kidneys by increasing the fecal excretion of heavy metals [41]. A previous study also showed that OA protected against Cd-induced tissue injuries in mice [42]. This evidence clearly indicates that OA is effective in Cd detoxification, as well as in reducing Cd accumulation and facilitating Cd excretion.

### 3.2. OA Facilitated Cd Excretion by Increasing the Abundance of Gut Burkholderia

The gut microbiota, a vital micro-ecosystem in the human body, plays an essential role in the high-fat-diet-mediated detoxification of heavy metals, and positively correlates with the excretion of heavy metals in mice [41]. From earlier findings, Cd exposure decreased the Alpha indices of the gut microbiome, reducing the microbial community’s richness and diversity [43,44]. In this study, orally administered OA significantly restored the diversity and abundance of gut microbiota, particularly the *Burkholderia* genus. As a type species for the genus *Burkholderia, B. cepacia* was well-protected by OA under Cd stress. Likewise, the OA-related synthetic gene *OLE1* confers Cd resistance and alleviates Cd-induced damage [45,46]. Actually, *B. cepacia*, a Gram-negative and aerobic bacterial strain, has been reported to improve plant growth under Cd stress, showing a high Cd accumulation capacity in Cd-contaminating environments [30,47,48]. A similar result was observed in a previous report, in which *Lactobacillus plantarum* CCFM8610 effectively decreased the intestinal Cd absorption and Cd accumulation in tissues [49]. Thus, the evidence validates that OA may confer Cd resistance and increase the abundance of *Burkholderia* in the gut, contributing to facilitated Cd excretion.

### 3.3. The Cd Absorptive Ability of B. cepacia and Its EPSs

Bacteria are usually considered to have an extensive capacity to absorb metal ions from solutions [50]. Bacteria are used for the bioremediation of Cd-polluted water via different surface interaction mechanisms, including complexation and inorganic microprecipitation [51]. Most Cd is adsorbed onto the cell surface or the biofilm [52]. As the key elements of biofilm, EPSs play an important role in the biosorption of heavy metals [53,54]. In this study, oral administration of *B. cepacia* significantly reduced the Cd accumulation in Cd-exposed mice. More importantly, high Cd levels were detected on the surface of *B. cepacia*, which was confirmed via SEM, TEM, and EDS analyses. This result is in line with an earlier report, in which extracellular bioaccumulation was identified to be primarily responsible for Cd biosorption [31].

The early evidence also indicates that the increasing extracellular accumulation of Cd might be due to the production of EPSs [55]. EPSs exhibit strong metal binding and complexation potential, which effectively influences the mobility and bioavailability of heavy metal in the environment [54]. EPSs are secreted to participate in this process when exposed to Cd [31]. In the current study, the EPS production in *B. cepacia* gradually increased with the exposure concentration of Cd in a dose-dependent manner. Higher levels of EPSs were produced for the high-exposure concentrations of Cd, and a higher capacity for Cd adsorption was displayed. A similar finding was observed previously, in which the EPS production of *B. cenocepacia* YG-3 increased with the addition of Cd (between 100 and 500 mg/L) [56]. Consequently, the data indicated that *B. cepacia* possesses remarkable Cd absorption ability, removing Cd from the cellular environment by producing EPSs in response to Cd.

### 3.4. The Cd Absorption Mechanism of the EPS of B. cepacia and Its Sulfhydryl Group

As is well known, bacteria possess the capacity to absorb metal ions [50]. Bacteria were reported in the sorption of Cd via various surface interaction mechanisms [57]. The surface binding of metals is a kind of metal complexation reaction with functional groups of biomolecules [58]. *Klebsiella aerogenes* was reported to absorb Cd through biosynthesizing insoluble cadmium sulfide particles on the cell surface [59]. In this study, an abundance of insoluble Cd particles was observed on the surface of *B. cepacia* after being exposed to Cd. Some researchers also found that the extracellular sorption of Cd just was mainly attributed to functional groups on the cell wall [60]. A previous study reported that the extracellular sorption of Cd in *Pseudomonas putida* might be due to the production of EPSs [55].

The enhanced Cd-adsorption capacity of EPSs may be attributed to the increased adsorption sites or functional groups. Functional groups on the outer membrane with high affinity to metals were found to play a major role in the binding to Cd [61]. Functional groups of EPSs, such as phosphate and sulfhydryl groups, were reported to have a strong affinity for Cd [62,63]. In this study, the FTIR analysis of the EPSs showed that the peak at 2355 cm^−1^ (-SH) increased in the presence of Cd. A similar finding was observed in a previous report, in which the interaction between Cd and the EPSs of *Pseudomonas aeruginosa* produced a significant increase in the FTIR peak at 2365.09 cm^−1^ (-SH) [64]. -SH was deemed to be a soft base ligand capable of binding with Pb strongly by forming covalent bonds [65]. More importantly, Cd has a high affinity for sulfhydryl groups [66]. In a previous study, a significant increase was observed in the content of sulfhydryl groups in *Tetraselmis suecica* cultures with the increase in the exposure concentration of Cd [67]. Notably, the increased intracellular Cd in *T. suecica* correlated with the increasing sulfhydryl groups [67]. The detection of -SH stretching upon the interaction with Cd confirmed the involvement of the sulfhydryl functional group in Cd binding. The current study confirms that the total sulfhydryl group (-SH) content of the EPSs of *B. cepacia* significantly increases after Cd exposure.

## 4. Materials and Methods

### 4.1. Materials and Reagents

Cadmium chloride (CdCl_2_, 98%) was purchased from Chengdu Huaxia Chemical Reagent (Chengdu, China). *Burkholderia Cepacia* (ATCC 25416) was purchased from Beijing Beina Chuanglian Biotechnology Institute.

### 4.2. Animals and Experimental Design

A total of 48 mice (8 weeks) were purchased from SPF (Beijing, China) Biotechnology Co., Ltd. (SCXK2019-0010). All mice were fed with the standard commercial rat food and kept in cages under the condition of 12 h light/dark cycles, and they were given free access to food and water.

To investigate the attenuation of various fatty acids against Cd, 30 SPF mice were randomly divided into 5 groups (n = 6). In the control group, the experimental mice received normal drinking water without Cd. For the Cd-exposed group, mice were given drinking water containing 100 μmol/L of CdCl_2_. The mice in the fatty acid-treated groups were given drinking water containing 100 μmol/L of CdCl_2_ and followed by intragastric administration containing 40 μmol/g/d of each fatty acids. Treatment was continued for 7 days.

To investigate the attenuation of *B. cepacia* against Cd toxicity, 18 SPF mice were randomly divided into 3 groups (n = 6). In the control group, mice received normal drinking water without Cd. For the Cd group, mice were exposed to the drinking water containing 100 μmol/L of CdCl_2_. In the Cd + *B. cepacia* group, mice were given drinking water with 100 μmol/L of CdCl_2_ and an intragastric administration containing 1×10^9^ CFU of *B. cepacia*. Treatment was also continued for 7 days. The above doses of Cd and *B. cepacia* were in accordance with the study of Qixiao et al. [68]

Animals were given a standard laboratory diet and water. The experiment was approved by the Animal Ethics Committee of Guangdong Ocean University (No.: GDOU-LAE-2021-020).

### 4.3. Determination of Cd Contents in Feces and Tissues

The feces samples from each group were collected every day. After sacrifice, small sections of tissues (gut, heart, liver, spleen, lung, and kidneys) of mice were collected and stored at −80 °C. The feces and tissue samples were crushed, ground evenly, and digested with HNO_3_ using a graphite digestion system (GDANA DS-360, Guangzhou, China) [11]. Cd content was measured by an inductive coupled plasma mass spectrometer (ICP-MS, ThermoFisher, ICAPQ, Germany) according to the manufacturer’s instructions. All analyses were run in triplicate.

### 4.4. 16S rRNA Gene Sequencing of the Gut Microbiota

16S rRNA gene sequencing of the gut microbiota was performed as previously described [69]. Total DNA of fresh fecal samples in each group were extracted with the PowerMax (stool/soil) DNA isolation kit (MoBioLaboratories, Carlsbad, CA, USA) according to the manufacturer’s instructions. The NanoDrop ND-1000 spectrophotometer (Thermo Fisher Scientific, Waltham, MA, USA) and agarose gel electrophoresis were used for quantity and quality measurement, respectively. The 16S rRNA gene sequencing was amplified using the forward primer 515F (5′-GTGCCAGCMGCCGCGGTAA-3′) and the reverse primer 806R (5′-GGACTACHVGGGT WTCTAAT-3′) targeting the V4 region. The PCR products were purified with AMPure XP Beads (Beckman Coulter, Indianapolis, IN, USA), and quantified with the PicoGreen dsDNA Assay Kit (Invitrogen, Carlsbad, CA, USA) before paired-end 2 × 150 bp sequencing using the Illlumina Novaseq 6000 platform at GUHE Info technology Co., Ltd. (Hangzhou, China)

Raw tags were processed using Vsearch v2.4.4 after removing the Barcode and primer sequence for obtaining effective tags through quality filtration. Sequencing data were processed using Quantitative Insights Into Microbial Ecology (QIIME, v3.2.0), clustering high-quality reads into operational taxonomic units (OTUs) at the 97% identity level. QIIME and R packages (v3.2.0) were used for the sequence data analyses.

### 4.5. Cd Sensitivity Assay of B. cepacia by the Growth of B. cepacia Cells

The *B. cepacia* was cultured in Luria-Bertani (LB) broth for 24 h at 37 °C with shaking at 130 r/min. *B. cepacia* was cultivated in LB containing 0, 100, 200, and 500 mg/L of Cd levels or 2 mmol/L of OA, and the optical density (OD) was measured at 600 nm to evaluate Cd sensitivity.

### 4.6. Determination of Cd Contents Accumulated Intracellularly, Extracellularly, and in the Supernatant of B. cepacia

The Cd contents accumulated intracellularly, extracellularly, and in the supernatant of *B. cepacia* were determined as previously described [31]. The *B. cepacia* cells were cultivated in LB broth containing 0, 100, 200, and 500 mg/L of CdCl_2_ for 1 to 4 days. The supernatant was obtained by centrifugation at 12,000× *g* at 4 °C for 15 min and then filtrated through a 0.22 μm cellulose acetate membrane. The remaining bacterial pellets were washed with 0.1 mol/L of EDTA-2 Na for 3 h and then centrifuged to collect Cd bound to the cell surface. The residual pellets were digested with HNO_3_ to determine the Cd absorbed in the cells. Cd contents in the supernatant, on the cell surface, and within the cells were determined by ICP-MS. All experiments were run in triplicate.

### 4.7. EPS Extraction

EPS extraction was performed as previously described [48]. The *B. cepacia* was cultured aerobically in LB broth for 72 h. First, the bacteria supernatant after exposure with the different CdCl_2_ was separately collected by centrifugation at 5000× *g* at 4 °C for 10 min. Secondly, the same volume of cold ethanol was added into the supernatant and kept at −20 °C overnight for precipitation of EPSs. Then, the EPS pellet was collected by centrifugation at 9000× *g* at 4 °C for 15 min and further dissolved in distilled water and then was dialyzed at 40 °C for 48 h. Finally, the pure EPS was obtained by lyophilization.

### 4.8. Electron Microscopy Analysis

The cell precipitate was collected by centrifugation at 4500× *g* at 4 °C for 10 min, and slightly washed with PBS aiming to remove medium. It was then fixed in 2.5% glutaraldehyde solution at 4 °C overnight. Then, cells were dehydrated with ethanol gradient and finally lyophilized for scanning electron microscopy (SEM, JSM-7610F) observation equipped with energy-dispersive spectroscopy (EDS, NORAN System 7). For transmission electron microscopy (TEM, TECNAI G2 20 TWIN) observation, the bacteria were prepared the same as for SEM from collection to dehydration steps, then embedded and sectioned into ultra-thin sections, and finally stained for microscopical observation [56].

### 4.9. Fourier Transform Infrared Spectrometer (FTIR) Assay

The EPS of *B. cepacia* was freeze-dried and mixed with KBr at a 1:100 mass ratio. FTIR spectroscopy (TENSOR 27, BRUKER OPTICS) was employed to examine the changes in structural vibration information of EPSs and the interaction with Cd under different Cd concentrations at 4 cm^−1^ spectral resolution within 4000–500 cm^−1^ [70].

### 4.10. Statistical Analyses

The data are expressed as the mean ± standard deviation (SD). All statistical analyses were performed by using the Graphpad Prism version 8.0. The comparison between groups were performed using Student’s *t* test. A *p* value of <0.05 was considered to indicate statistical significance.

## 5. Conclusions

The data from this study confirm that OA is the most effective of the three free fatty acids in reducing Cd accumulation through facilitating Cd excretion in mice. OA facilitates Cd excretion by increasing the abundance of the *Burkholderia* genus in the gut microbiota. In addition, as the type species for the *Burkholderia* genus, *B. cepacia* conferred Cd tolerance by OA. More importantly, *B. cepacia* facilitated the Cd excretion through secreting EPSs with a high sulfhydryl group (-SH) content following Cd exposure. Finally, OA is a potentially suitable supplemental therapy to reduce Cd accumulation in the body.

## Data Availability

The data presented in this study are available on request from the corresponding author.
